# A case report of immunosuppression-related Kaposi’s sarcoma after autologous stem cell transplantation

**DOI:** 10.1186/s13104-016-1991-9

**Published:** 2016-03-24

**Authors:** Bert Heyrman, Ann De Becker, Rik Schots

**Affiliations:** Department of Clinical Hematology, UZ Brussel-VUB, Laarbeeklaan 101, 1090 Jette-Brussels, Belgium

**Keywords:** Kaposi’s sarcoma, Autologous stem cell transplantation

## Abstract

**Background:**

Kaposi’s sarcoma (KS) is a tumor formed by angioproliferations driven by Human herpes virus 8 also known as Kaposi’s sarcoma-associated herpes virus (KSHV). It is best known as an acquired immune deficiency syndrome (AIDS) defining illness that may be fatal. There are only a few reports of KS after hematopoietic cell transplantation (HCT). This is the first case describing the disappearance of KS with immune recovery after autologous HCT.

**Case presentation:**

We present the case of a 61-year-old male heterosexual patient of Moroccan origin treated for primary mediastinal non-Hodgkin lymphoma. Because of refractory disease he received multiple lines of chemotherapy prior to autologous HCT. After the second course of low-dose bis-chloroethylnitrosourea, etoposide, cytarabine, melphalan (BEAM) the patient developed several round blue skin lesions. A biopsy was performed, showing many small vessels and positive immune histochemical staining for Human herpes virus 8 (HHV-8), confirming diagnosis of KS. Human immunodeficiency virus testing was negative and work-up showed that there were no visceral lesions. When KS are limited to the skin, prognosis is usually better. The extensive chemotherapy resulted in an important immunosuppression; on day 105 after autologous HCT CD4^+^ count was 82/mm^3^. Since KS were limited to the skin and attributed to severe immune suppression a watchful waiting strategy was adopted even though in the first months after autologous HCT new skin lesions appeared. With immune recovery (CD4^+^ count > 200/mm^3^) 277 days after transplant, skin lesions faded.

**Conclusion:**

Kaposi’s sarcoma remains a rare tumor that should be thought of in any patient whose immunity is down. If immune recovery is expected and disease is limited to the skin, a watchful waiting strategy can be more rewarding than intensive chemotherapy.

## Background

Kaposi’s sarcoma (KS) is a tumor characterized by mucocutaneous and visceral angioproliferations, first described by Moritz Kaposi in 1872 [1]. Today four clinical variants of KS are known [1] with similar histologic findings but different sites of involvement and rates of progression. Classic Kaposi’s sarcoma, primarily found in Eastern European and Mediterranean men (male to female ratio 15:1) is mostly limited to the skin of hands and feet and follows an indolent course. Endemic KS occurs in Africa independent of Human immunodeficiency virus (HIV) infection. In children progression is fulminant and outcome fatal whereas in adults presentation is similar to classic KS. Immunosuppression- or transplantation-associated KS occurs in organ-transplant recipients and patients receiving immunosuppressive therapy for other medical conditions. This type tends to be aggressive, involving lymph nodes, mucosa and visceral organs, sometimes in the absence of skin lesions. Epidemic or acquired immune deficiency syndrome (AIDS) associated Kaposi sarcoma is the best known variant and is commonly known to be an AIDS defining illness in HIV patients. It is feared for its aggressive and frequently fatal outcome.

Only 13 cases of KS after hematopoietic cell transplantation (HCT) have been described: 11 after allogeneic and two after autologous HCT [2–14]. The disease was lethal in 3/11 allogeneic and 1/2 autologous HCT. In adults, skin only was the dominant clinical presentation. All three pediatric cases had visceral involvement, resulting in two fatalities (after either allogeneic or autologous HCT). The two cases described after autologous HCT had visceral involvement. The adult case, while in complete remission for his hematological malignancy, died due to disseminated KS. The pediatric case responded to recombinant alpha-interferon.

The driving agent for the proliferative growth is Kaposi’s sarcoma-associated herpes virus (KSHV) also known as Human herpes virus 8 (HHV-8), a group 1 carcinogenic agent according to the International Agency for Research on Cancer [15]. Apart from KS, HHV-8 is the underlying cause of lymphoproliferative disorders such as primary effusion lymphoma [16] and multicentric Castleman disease [17]. Seroprevalence rates are low in Western countries and higher in certain geographic areas (the Mediterranean area and Africa) [18, 19]. Human herpes virus 8 prevalence is high in men with homosexual activity, apart from HIV infection and in migrants from endemic regions [17]. Saliva is believed to be the major transmission vehicle for horizontal transmission [20, 21].

Genes encoded by KSHV can induce cellular proliferation and prevent apoptosis causing a transformative effect on endothelial cells. In transplant recipients nonmalignant pathologic events as cytopenias and acute hepatitis syndromes can be provoked by KSHV [22]. One study however, conducted to investigate HHV-8 seroprevalence after allogeneic bone marrow transplantation (BMT) did not find any association between presence of antibodies directed against HHV-8 latent nuclear antigen before or after transplantation, chronic graft versus host disease, or overall BMT survival [23]. More data are needed to support this finding.

In general the level of immunosuppression and the extent of disease determine the course of KS. Visceral KS is characterized by rapid spread and risk of major bleeding with often fatal outcome. Skin limited KS typically follows an indolent course [24] and is more an aesthetic problem lowering self-esteem of HIV patients. In solid organ transplant patients 1 year survival rate is at 90 % for cutaneous disease and 70 % for visceral forms [25].

Immune recovery is generally defined as a rise in CD4^+^ count above 200/mm^3^ and was described to be associated with a gradual disappearance of KS in HIV patients [26, 27]. Since the introduction of HAART as a successful HIV treatment resulting in less prolonged immune deficiency, the incidence of KS has decreased substantially [28, 29].

Treatment intent and strategy differ depending on location and spread of disease. Tapering of dose in patients receiving immunosuppressive medication is an option. In disease limited to skin only, surgical excision or electro-chemotherapy is the most preferable approach, radiotherapy appears to be abandoned due to local side effects (edema and ulcerations) [22]. In solid organ transplant patients intensive therapy is required in case of persistent functional disability or life-threatening disease [30]. Possible options are the use of interferon-alpha or chemotherapy using doxorubicin in monotherapy or vincristine, etoposide and bleomycin in monotherapy or in combination with anthracyclines [31]. The use of antiviral treatment (cidofovir, foscarnet, ganciclovir or valganciclovir) is considered without benefit [32, 33]. Since neoplastic KS spindle cells harbor a latent HHV-8 infection eradication is not possible [34–36]. One could start antiviral therapy in cases of aggressive disseminated KS with high HHV-8 viremia, aiming to at least resolve cytopenias. Novel targeted therapies are currently under investigation. Imatinib a tyrosine kinase inhibitor of c-Kit, expressed by KS tumor cells, showed promising results in a phase 2 study conducted in AIDS-related KS patients [37].

Here we report the rise and fall of Kaposi sarcoma of the skin coinciding with immunosuppression and reconstitution after autologous HCT in a 61-year-old patient of Moroccan origin treated for primary mediastinal non-Hodgkin lymphoma.

## Case presentation

### Case history

A 61-year-old heterosexual male of Moroccan origin was diagnosed with primary mediastinal non-Hodgkin lymphoma. Other than hypertension treated with amlodipine he had no medical history. Viral serology prior to starting therapy showed previous hepatitis B infection, HIV screening was negative.

**Fig. 1:**
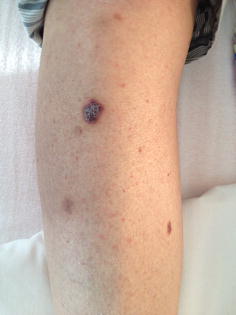
Kaposi sarcoma on the right leg

**Fig. 2:**
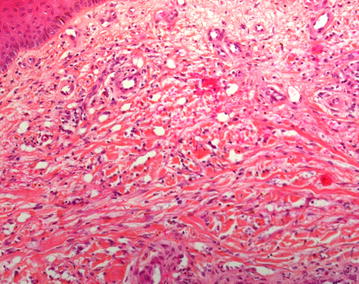
Endothelial swelling [Skin biopsy, HES (hematoxylin, eosin, saffran) routine staining, 200×]

**Fig. 3:**
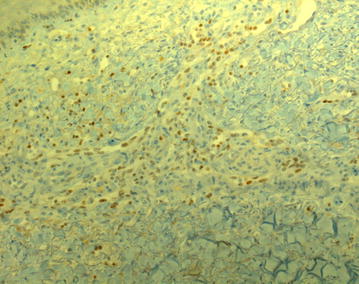
HHV-8 positivity (Skin biopsy, immune histochemical staining HHV-8, 200×)

The patient was refractory to rituximab, cyclophosphamide, doxorubicin, vincristine and prednisone (R-CHOP). Second line therapy with rituximab, dexamethasone, cytarabine and cisplatin (R-DHAP) had to be abandoned after one cycle because of invalidating tinnitus. Rituximab, ifosfamide, carboplatin and etoposide (R-ICE) was started with an increase in tumor activity and volume on positron emission tomography (PET)/computed tomography (CT) after the first cycle. In August 2013 the patient was hospitalized for high dose cyclophosphamide for stem cell mobilization. Treatment continued with low-dose BEAM (bis-chloroethylnitrosourea 60 mg/m^2^ on day 1, etoposide 15 mg/m^2^ on day 2,3,4 and 5, cytarabine 2 × 100 mg/m^2^ on day 2,3,4 and 5, and melphalan 30 mg/m^2^ on day 6) followed by autologous stem cell rescue, resulting in a partial response. We repeated low-dose BEAM followed by stem cell infusion and obtained stable disease on PET/CT. After the second cycle we noticed blue, round lesions on the left forearm and both legs, slightly elevated and sensitive with pressure (Fig. 1). Skin biopsies were taken. Microscopy showed a normal epidermis, but the dermis showed many small vessels in clusters with swollen endothelial cells (Fig. 2). Immune-histochemical staining was positive for HHV-8 (Fig. 3) and negative for cytomegalovirus (CMV). The diagnosis of Kaposi’s sarcoma of the skin was made. Serology confirmed a negative HIV status. Review of the CT scans showed no evidence of visceral sarcomas. After 1 week of ganciclovir because of a positive polymerase chain reaction (PCR) for CMV, treatment continued with full dose BEAM (bis-chloroethylnitrosourea 300 mg/m^2^ on day 1, etoposide 200 mg/m^2^ day 2–5, cytarabine 2 × 200 mg/m^2^ day 2–5, and melphalan 140 mg/m^2^ day 6) and autologous stem cell transplantation. During hospitalization no new skin lesions appeared.

Two months later more skin lesions appeared on the lower legs. PET/CT at that moment showed a fibrotic lesion in the anterior mediastinum with volume decrease and complete resolution of metabolic activity. The patient was consolidated with 40 Gy radiotherapy. During this treatment additional lesions appeared on the lower legs, his general condition was cumbersome. A new biopsy confirmed the previous diagnosis of Kaposi’s sarcoma. CD4^+^ count at that time, 3 months after the last HCT was at 82/mm^3^. Later, the spreading of KS stopped and on day 182 after HCT CD4^+^ count rose to 156/mm^3^.

**Fig. 4:**
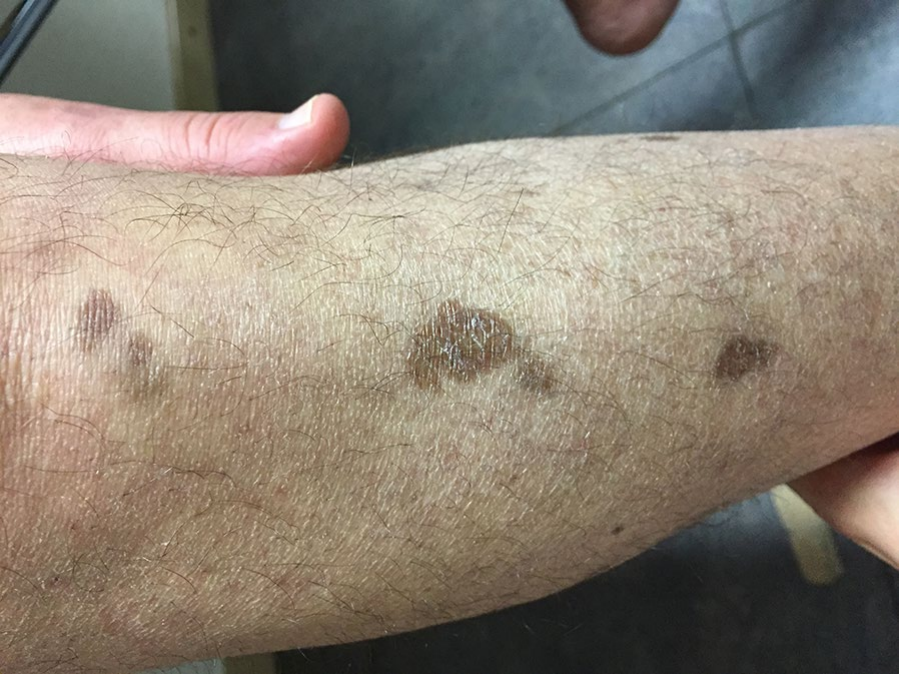
Fading of Kaposi sarcoma on the right leg

Nine months after transplantation, counting 227 CD4^+^/mm^3^ in peripheral blood, skin lesions gradually disappeared (Fig. 4). A new CT scan confirmed complete response status of the lymphoma.

## Discussion

In this case study we report a patient with dermal Kaposi’s sarcomas associated with HHV-8 appearing shortly after autologous stem cell transplantation. Spread was limited to the skin; initially the left forearm and lower legs, later some abdominal skin lesions appeared. HHV-8 serology prior to transplantation was not known and not a routine examination in our center. With a negative history in male sexual contact, we consider our patient from Moroccan origin to be an endemic carrier.

Several lines of intensive chemotherapy followed by autologous HCT is considered an aggressive form of immune suppression, without immune suppressive therapy in relation to transplant. The patient‘s immune deficient state was confirmed by a low CD4^+^ count of 82/mm^3^ 105 days after the last HCT. On day 182 CD4^+^ count rose to 156/mm^3^ and to 227/mm^3^ on day 277 after HCT, coinciding with a remarkable decrease in skin lesions. This observation supports that KS in this case was related to a low CD4^+^ count and not a case of classic KS.

In our case there was only limited disease of the skin without aesthetic concern. We focused on the initial disease for further treatment, as this was considered an intense treatment option for KS as well. However intense chemotherapy using full dose BEAM did not ameliorate his original lesions. When new KS were formed, we decided to watch and wait for immune recovery. It was not until CD4^+^ count rose to 227/mm^3^ that the lesions clearly faded.

## Conclusions

This is the third case of appearance of KS and the first describing resolution of Kaposi’s sarcoma with CD4^+^ cell recovery after autologous HCT. Kaposi’s sarcoma remains a rare tumor that should be thought of in any patient whose immunity is down. If immune recovery is expected and disease is limited to the skin, a watchful waiting strategy can be more rewarding than intensive chemotherapy.

## Consent

Written informed consent was obtained from the patient for publication of this case report and any accompanying images.

## Abbreviations

KS: Kaposi’s sarcoma
HHV-8: human herpes virus 8
HCT: hematopoietic cell transplantation
CMV: cytomegalovirus
HIV: human immunodeficiency virus
AIDS: acquired immune deficiency syndrome
PET: positron emission tomography
CT: computed tomography
R-CHOP: rituximab cyclophosphamide doxorubicin vincristine prednisone
R-DHAP: rituximab dexamethasone cytarabine cisplatin
R-ICE: rituximab ifosfamide carboplatin etoposide
BEAM: bis-chloroethylnitrosourea etoposide cytosar melphalan
